# CellNOptR: a flexible toolkit to train protein signaling networks to data using multiple logic formalisms

**DOI:** 10.1186/1752-0509-6-133

**Published:** 2012-10-18

**Authors:** Camille Terfve, Thomas Cokelaer, David Henriques, Aidan MacNamara, Emanuel Goncalves, Melody K Morris, Martijn van Iersel, Douglas A Lauffenburger, Julio Saez-Rodriguez

**Affiliations:** 1 European Bioinformatics Institute (EMBL-EBI), Wellcome Trust Genome Campus, Cambridge CB10 1SD, UK; 2 Biological Engineering Department, Massachusetts Institute of Technology, Cambridge, MA, USA

**Keywords:** Signaling networks, Systems biology, Phosphoproteomics, Logic modeling, Perturbation data

## Abstract

**Background:**

Cells process signals using complex and dynamic networks. Studying how this is performed in a context and cell type specific way is essential to understand signaling both in physiological and diseased situations. Context-specific medium/high throughput proteomic data measured upon perturbation is now relatively easy to obtain but formalisms that can take advantage of these features to build models of signaling are still comparatively scarce.

**Results:**

Here we present *CellNOptR*, an open-source R software package for building predictive logic models of signaling networks by training networks derived from prior knowledge to signaling (typically phosphoproteomic) data. *CellNOptR* features different logic formalisms, from Boolean models to differential equations, in a common framework. These different logic model representations accommodate state and time values with increasing levels of detail. We provide in addition an interface via Cytoscape (*CytoCopteR*) to facilitate use and integration with Cytoscape network-based capabilities.

**Conclusions:**

Models generated with this pipeline have two key features. First, they are constrained by prior knowledge about the network but trained to data. They are therefore context and cell line specific, which results in enhanced predictive and mechanistic insights. Second, they can be built using different logic formalisms depending on the richness of the available data. Models built with *CellNOptR* are useful tools to understand how signals are processed by cells and how this is altered in disease. They can be used to predict the effect of perturbations (individual or in combinations), and potentially to engineer therapies that have differential effects/side effects depending on the cell type or context.

## Background

Cells receive and interpret information through complex signaling networks. The correct processing of signals is essential and frequently altered in diseases [1-3]. Signaling networks arise from the highly dynamic and context specific assembly of a large variety of molecular species [3]. It is increasingly recognised that including these features is essential to take our understanding of the functionality of signaling pathways to the next level [4]. Knowledge about signaling networks has accumulated over the years in databases and literature [5-10]. The vast majority of this information is static and not context-specific, and provides limited insight into the system’s response to perturbations such as ligand stimulation or drug treatments [4,11-13].

Gathering medium to high-throughput signaling data is becoming more feasible as proteomic technologies are getting more mature [14]. Perturbation data (such as chemical inhibitors, stimuli, knock-downs, etc) can be used to generate network models using reverse engineering methods [14-16]. These methods typically consider all possible topologies. Thus, they require large amounts of data and scale-up poorly. Furthermore, the resulting networks are limited to interactions between perturbed and measured nodes. These are typically only a subset of the nodes involved in a pathway. Therefore, such models are not as biologically interpretable as a networks based on prior knowledge from literature and other sources.

We recently introduced a method that integrates literature and perturbation data to overcome the shortcomings of both [11]. By training prior knowledge networks (PKNs) against experimental data, this method produces models shown to achieve significantly better predictive power than untrained models. The model building process is implemented through the use of a logic formalism. Logic models have the ability to capture cause-effect relationships while staying conceptually and computationally simple, thereby allowing for appreciable scalability [17]. In its simplest implementation, Boolean logic [18], species are described as either ON or OFF. Relationships between species are described using logic gates that specify the state of each node given the state of its parents [19]. This captures dependencies between components in a system without the requirement of detailed mechanistic knowledge [17]. Logic models have been shown to be useful tools to study signaling and regulatory networks [17,19-23]. A number of tools exist to manipulate, create and simulate such models [24-32], and the approach described in [11] complements them by automatically generating models trained to data. This allows researchers to generate models of signaling that can answer biological questions in their specific system of interest. However, the method in [11] was limited to Boolean logic steady state representation of the system under investigation, and was only available in a closed-source package for the MATLAB environment.

We present here a tool that implements the methods in [11] in an open source R/Bioconductor package (*CellNOptR*). *CellNOptR* extends the methods presented in [11] to various published and unpublished logic formalisms through a suite of additional R packages that are integrated with the *CellNOptR* package. These logic formalisms include Boolean steady-state, Boolean multiple steady-state, Boolean discrete time, steady-state fuzzy logic and logic-derived ordinary differential equation (ODE) representations of the system. This set of packages forms an integrated, open source, robust and easily extendable platform for training logic models of signaling networks. *CellNOptR* can also be used via a graphical user interface through the Cytoscape plugin *CytoCopteR*. We illustrate the application of *CellNOptR* to a simulated example showing the advantages of having multiple logic formalisms available. We then show how the package can be used to study early and late response of a human hepatocellular carcinoma cell line to several cytokines.

## Implementation

### The *CellNOptR* approach

**Figure 1:**
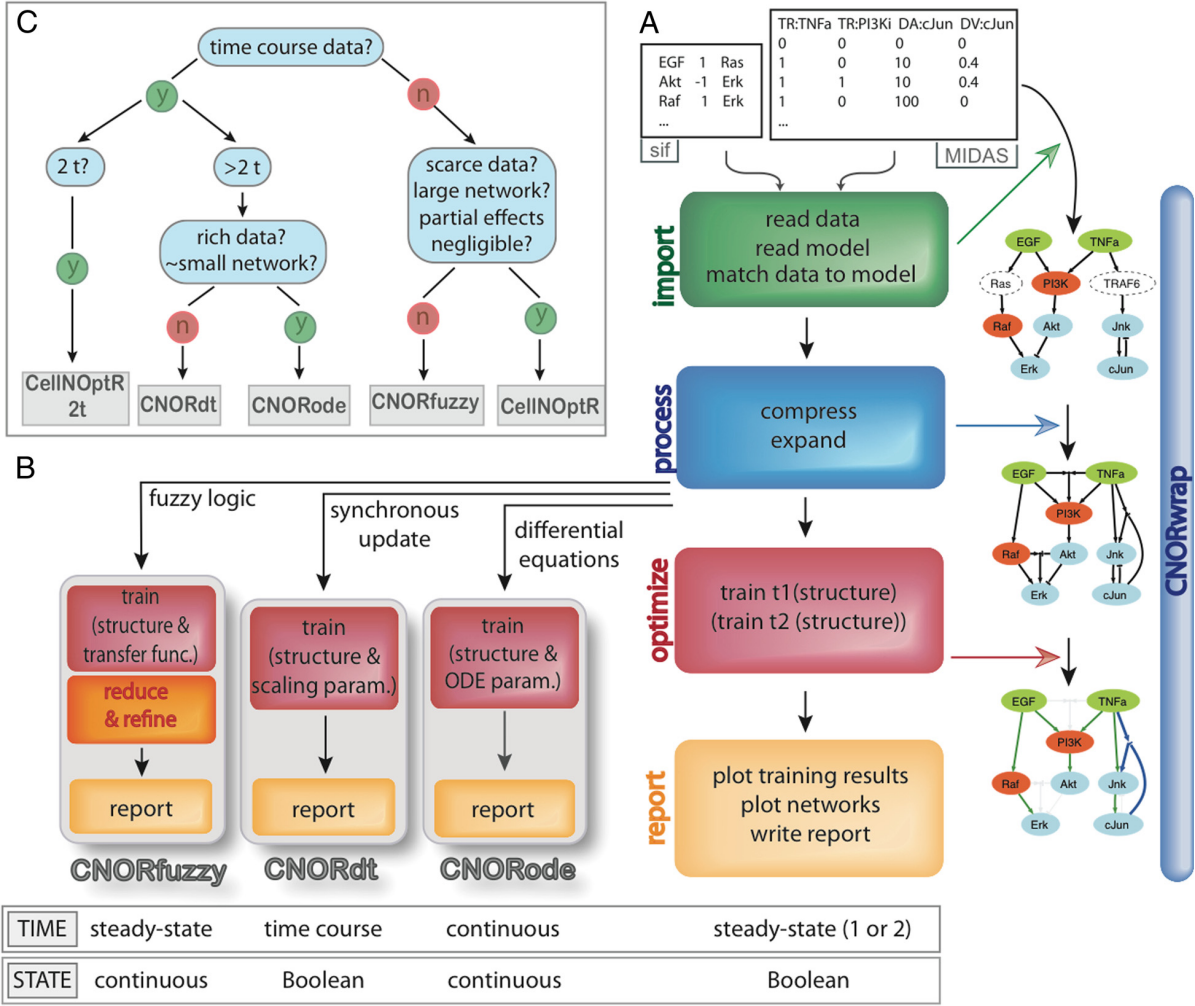
**The *CellNOptR *framework. A**. A *CellNOptR* analysis takes as input 2 text files: (1) a Prior Knowledge Network (PKN) as a SIF file [39], (2) a dataset in the MIDAS format ( [34], see Figure 4). The package then maps the data onto the PKN, processes the network and trains the resulting model. *CellNOptR* outputs a series of HTML pages containing the summary of the analysis, hyperlinked to diagnostic graphs, and the trained networks. Multiple logic formalisms can be used for the training. The *CellNOptR* package implements most of the workflow and the simplest Boolean logic steady-state (1 or 2) approach. **B**. Only steps that are specific to a particular logic formalism are coded in add-on packages. *CNORfuzzy* implements a constrained fuzzy logic steady-state approach [35]. *CNORdt* fits time course data using a Boolean representation of the states of nodes and a synchronous update simulation scheme. *CNORode* fits detailed time course data by deriving and training continuous logic-based ordinary differential equations. **C**. The choice of a logic formalism depends on the data at hand and the modeling goals: with no time course data, the user can choose between the two steady-state implementations (*CNORfuzzy* and *CellNOptR*) based on the size of the network, richness of data and suspected impact of partial effects. If very limited time course data is available, users can use the Boolean 2 steady-states implementation in *CellNOptR*. With detailed time course data, one can choose between the Boolean discrete time implementation in *CNORdt* and the continuous ODE based implementation in *CNORode*, mainly based on the complexity of the network and the richness of the data. For the networks, the following color conventions are used: for nodes: green=stimulated, red=inhibited, blue=measured, dashed=compressed; edges (referring to the optimised model): green=present at time 1, blue=present at time 2, grey=absent, dashed edge=compressed.

*CellNOptR* (for Cellular Network OptimizeR) implements the method introduced in [11] in the R language, as a Bioconductor [33] package. This method derives a Boolean logic model from a “prior knowledge network” (PKN, i.e. a network obtained from literature or expert knowledge) and trains it against perturbation data. A *CellNOptR* analysis comprises the following steps (see Figure 1): (i) import of the network and data, (ii) processing of the network, (iii) training, and (iv) reporting the results of the analysis.

### Import of network and data

*CellNOptR* takes as input two flat text files. The first one is a prior knowledge network (PKN) describing signed and directed interactions between proteins as a graph (currently Simple Interaction File (SIF) format, which can be opened in Cytoscape). The second file contains biochemical data relating to the changes in the modification state (typically phosphorylation) of proteins following stimulation under various conditions. By “conditions” we refer to combinations of stimuli and inhibitors targeting nodes in the network. This data is represented in the simple tabular MIDAS (Minimum Information for Data Analysis in Systems biology) format introduced in [34] (see Figure 1).

The package then performs normalisation of the data for logic modeling, a feature described in [11] and previously implemented in a separate MATLAB package, DataRail [34]. Briefly, the data is normalised between 0 and 1 by computing a fold change relative to a control. This fold change is transformed through a Hill function and multiplied by a penalty for signals close to background. The penalty is the ratio of each value to the maximum measurement for the readout considered, transformed through a saturation function. It is important to note that the data is not discretized but just normalised between 0 and 1.

### Processing of the network

The network is converted into logic models for training with two pre-processing steps : (1) compression and (2) expansion. In the compression step, species that are neither measured nor perturbed are removed if the logical consistency of the network is not impaired, resulting in a simplified network for training. This step is performed because such nodes are not necessary for the correct training of the model. However, starting from a PKN facilitates: i) identifying and preserving nodes whose presence is necessary to maintain the logical consistency of the network, ii) mapping the trained model back onto the starting network (thereby preserving the interpretability) and iii) restricting the search to a set of interactions that are feasible based on prior knowledge. In the expansion step, interactions are converted into all possible logic gates. For example, if there is an edge from node B to A and node C to A, the following gates are created: (i) B AND C → A, (ii) B OR C → A, (iii) B → A, (iv) C → A. The rationale behind this step is that, although databases record a potentially functional interaction between A and B and A and C, it is rarely recorded whether these interactions are independent or not (i.e. B and C are both required to activate A, or only one of them), or even if any of them are active in the specific context under investigation. Therefore, *CellNOptR* generates all these options in the scaffold model (i.e. the compressed and expanded model that is used as a basis for optimisation) and uses the training to data to discriminate among them.

### Training

Next, the model is trained to data by searching for models (i.e. sub-models of the scaffold model, that include a subset of the edges) that minimize a bipartite optimisation function. The optimisation function weights the fit to data (deviation between data and the output of the Boolean logic model at steady state, in matched conditions) and model size, according to equation 1.

(1) $$\theta (P)=\theta_{f}(P)+\mathrm{\alpha .}\theta_{s}(P)$$

(2) $$\theta_{f}(P)=\frac{1}{n_{g}}\overset{s}{\underset{k=1}{\sum }}\overset{m}{\underset{l=1}{\sum }}\overset{n}{\underset{t=1}{\sum }}{\left(B_{k,l,t}^{M}-B_{k,l,t}^{E}\right)}^{2}$$

(3) $$\theta_{s}(P)=\frac{1}{v_{e}^{s}}\overset{r}{\underset{e=1}{\sum }}v_{e}P_{e}$$

In equation 1, *P* is a vector of length r (where r is the number of edges in the scaffold) with a 1 when an interaction is included and a 0 if it is not. *θ*_*f*_ (equation 2) is the mean squared deviation between model prediction (*B*^*M*^) and data (*B*^*E*^) across the *m* readouts, *n* time points and *s*experimental conditions (weighted by the total number of data points *n*_*g*_). *θ*_*s*_ (equation 3) penalises the model size by summing across the number of inputs (*v*_*e*_) of each edge selected in model P and dividing by the total number of inputs across all edges ( $v_{e}^{s}=\overset{r}{\underset{e=1}{\sum }}v_{e}$). *α*is a tunable parameter that balances the fit and size terms. The size penalty ensures that redundant or unnecessary edges are not included in the final model, such that simpler models are preferred over more complicated models if they explain the data equally well. Note that the data does not need to be discretized to compute the optimization function. Instead, the data can be normalized between 0 and 1 (see [11]), resulting in a penalty that depends on how close the normalized data is to the Boolean state predicted by the model. Thus, measurements that have intermediate values (and we are therefore less certain if they are ’on’ or ’off’) have a smaller weight on the penalty associated with a mismatch with the Boolean output of the model. The search through model space is performed using a built-in genetic algorithm. It is possible for the user to choose which edges they want to be included in the search (e.g. if part of the model structure is known with certainty, see the package vignette) but by default all edges are included in the search space. *CellNOptR* keeps track of all models explored during the search and reports a family of models within a tolerance (given by the user) of a value of the objective function *θ*. Indeed, multiple models with the same or very similar scores are typically found, which cannot be discriminated given the experimental evidence [35]. The choice of a tolerance level is non-trivial and depends largely on the experimental error. Indeed, as our confidence in the data increases, our tolerance regarding how closely the model have to reproduce the data decreases. Given a chosen tolerance level, CellNOptR reports, for each edge, the frequency of models within the tolerance limits that include the edge. This allows users to investigate the effect of the tolerance on the solution models, given the data at hand.

### Report

Finally, the results of the training are mapped to both the prior knowledge and the scaffold network. The information relating to the analysis run is then plotted, written to file and condensed in a HTML report hyperlinked to the various diagnostic plots. Networks are output in Graphviz DOT format as well as SIF files with corresponding attributes representing the status of nodes (compressed, measured, inhibited, etc.) and the frequency with which edges are selected in the family of solution models.

### Simulation variants

This general approach is extended through a series of add-on R packages that use parts of the *CellNOptR* method but differ in their ability to handle time course data with different levels of sophistication. *CellNOptR* implements the simplest logic framework, where a Boolean steady state approximation is used for simulation. *CellNOptR* also contains a Boolean 2 steady-states method, applicable when limited time resolved data is available and one wishes to capture mechanisms acting on different time scales. In addition, we offer three packages (see Figure 1) that plug into the *CellNOptR* approach and perform model training based on: (i) single pseudo-steady state data and a continuous representation of the state of nodes using a constrained fuzzy logic approach (*CNORfuzzy*) [35], (ii) coarse grained time resolved data and a discrete time simulation using a Boolean synchronous update scheme (*CNORdt*), and (iii) time course data and a continuous state and time simulation using ordinary differential equations derived from a logic model (*CNORode*) [36]. Functions in the add-on packages implement alternatives for core functions or additional steps whenever the handling of a more elaborate logic formalism requires it (see Figure 1).

### Languages and dependencies

All of our packages are written in R. In order to improve computational efficiency, the core of *CNORode* is written in C, using the standard R API as an interface. The simulation engine of *CNORode* uses the CVODES library. No compilation or code generation is required beyond the building of the package. For the model and parameter space search in *CNORode*, we give the option to use the R package genalg [37] or an R implementation of Scatter Search [38] (available as part of the package MEIGO, http://www.iim.csic.es/~gingproc/meigo.html). We provide a user-friendly evaluation function that allows users to easily plug in alternative search methods. We also provide Python wrappers so that CellNOpR can be run directly from Python (see http://www.cellnopt.org). Finally, we provide a graphical user interface via *CytoCopteR*, a Cytoscape [39] plugin. *CytoCopteR* uses the Cyrface (http://sourceforge.net/projects/cyrface/) Cytoscape plugin to interface with R and call our methods from Cytoscape.

## Results and discussion

### Various simulation schemes allow to capture different features of a system

**Figure 2:**
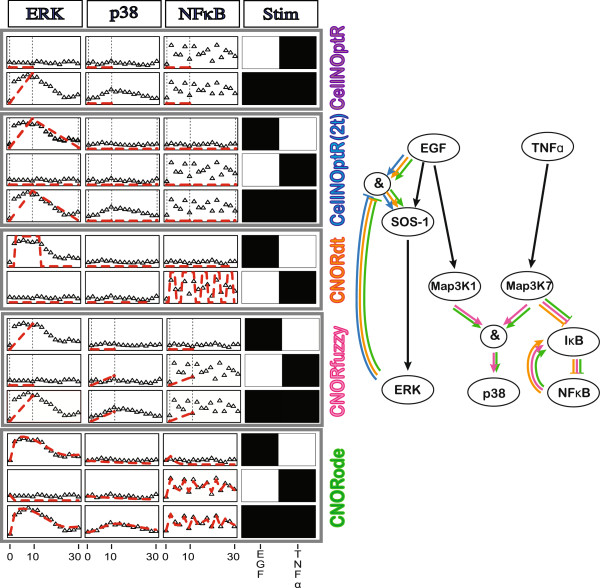
**Simulation schemes in the *CellNOptR *and add-ons packages. ***Adapted from*[40]. This network is a simplified version of a realistic toy example from [40], used to generate simulated data (triangles). We show a subset of the results of training this model to data using each of the logic formalisms available through our packages (dashed red lines). The model contains canonical pathways downstream of EGF and TNF*α*. The data includes: (i) a slow negative feedback from ERK to SOS-1 leading to a transient activation of ERK, (ii) a feedback from NF*κ*B to its inhibitor I*κ*B, leading to oscillations of NF*κ*B, and (iii) a partial activation of p38 under combined EGF-TNFa stimulations. The *CellNOptR* simulation scheme (Boolean, steady state) captures the activation of ERK upon EGF stimulation (black edges EGF - SOS-1 - ERK) but not its transient nature. The Boolean with two steady states version does capture the transient ERK activation (i.e. both the black path between EGF and ERK and the negative ’AND’ gate when both EGF and ERK are activated, blue edges) but not the NFkB oscillations and p38 partial activation. With the discrete time updating scheme with Boolean state from *CNORdt*, we capture both the transient activation of ERK and the NF*κ*B oscillations(orange edges) but not the partial activation of p38. *CNORfuzzy* implements a continuous representation of states but with a single steady state. Thus, it captures the partial activation of p38 (pink edges) but not the behaviors of ERK and NF*κ*B. *CNORode* is based on a continuous representation of both time and state, which captures the behaviors of ERK, p38 and NF*κ*B (green edges). Depending on the available data and the suspected behaviors to capture, different logic formalisms are more appropriate. Dashed lines=time points used for steady states. Color of model edges: black=captured by all approaches, blue = CellNOptR(2t), orange = CNORdt, pink=CNORfuzzy, green=CNORode.

Within the scope of logic models, various formalisms can be used to represent relationships between nodes and simulate a model. The choice of which logic formalism to use depends on the data set and the system to be modeled (see Figure 1). In the next sections, we present the different formalisms that are implemented in the *CellNOptR* framework. We illustrate their advantages and limitations on a simulated data set obtained from a realistic toy example from MacNamara et al. [40] that schematically represents the effect of Tumor Necrosis Factor *α*(TNF*α*) and Epidermal Growth Factor (EGF) on the canonical p38, ERK, and NF*κ*B pathways. The model used to generate the simulated data (see Figure 2) contains a slow negative feedback from ERK to SOS-1, leading to a transient activation of ERK. This could for example represent the expression of a phosphatase that dephosphorylates SOS-1 and whose expression depends on the activation of ERK. This model also includes a negative feedback from NF*κ*B to its inhibitor I*κ*B, leading to oscillations of NF*κ*B. This captures the observed oscillations of nuclear NF*κ*B, where the transcription factor is known to be maintained in the cytoplasm by its inhibitor whose expression is activated by NF*κ*B itself. Finally, a partial activation of p38 is observed when both EGF and TNF*α*stimulations are applied to the model.

### CellNOptR: Boolean logic at steady-state

The default *CellNOptR* method as described in [11] is based on a discrete representation of time and state. For the observed data, measurements are therefore acquired at the rest state as well as at a characteristic time after perturbation (pseudo-steady state). The states of nodes in the model are represented as Boolean values (ON/OFF). For simulation, we use a synchronous updating scheme until all nodes have reached a steady state. We compute the state of each node at time t+1 as a function of the state of its parents at time t (see equation 4), and check whether this new state is the same as the one at time t.

(4) $$\begin{array}{c} x_{i}(t+1)=B_{i}(x_{i1}(t),x_{i2}(t),\ldots ,x_{i1}(t))\;\;\;\varepsilon \{0,1\},\;\;i=1,2,\ldots ,N \end{array}$$

In equation 4, the state of each species *x*_*i*_ at time *t* + 1 is computed as a Boolean function B of the states at time t of all nodes *x*_*iN*_ upon which *x*_*i*_ depends. Equation 4 is applied simultaneously to all nodes in the model until all *x*_*i*_(*t* + 1) = *x*_*i*_(*t*) or a maximum number of iterations has been reached. Nodes that oscillate (because e.g. of a negative feedback loop, see below) never reach the steady state and are therefore penalized as mismatches to experimental data. As can be seen in Figure 2, this means that the basic *CellNOptR* method is unable to capture the Nf*κ*B oscillations, as well as the partial activation of p38. Because it only considers one time point, the model is also unable to capture the transient activation of ERK. Consequently, although it will detect an activating edge between SOS-1 and ERK, it will not detect the negative feedback between ERK and SOS-1. Nonetheless, provided that the pseudo-steady state time point is appropriately chosen, this very simple and computationally efficient approach captures most of the links in this network.

### CellNOptR(2t): Boolean logic at 2 steady-states

If, however, we wish to capture the transient activation of ERK, we can do so using a previously unpublished modification of the Boolean steady-state method which is available in *CellNOptR*. This modified version uses data collected at two separate time points (see Figure 2), which are assumed to represent logical pseudo steady states, resulting from mechanisms that operate at different time scales. For example, this method could be used to model immediate and fast receptor activation by post-translational modification followed by propagation of the signal and receptor desensitization depending on *de novo* protein expression. Assuming two different time scales allows us to train the model to the 2 pseudo-steady states independently, thereby keeping the method computationally efficient.

Using this method we first train the model using the data at the first time (*τ*_1_) point just as above. In a second training step, we assume that some edges only become active at the second time point (*τ*_2_), and therefore search through the space of edges not included in the optimal model at *τ*_1_. We simulate the model using the steady state of *τ*_1_ as an initial state, with the added constraint that nodes receiving the input of a *τ*_2_ edge are locked to the state defined by that edge. This is to avoid nodes in a negative feedback loop never reaching a Boolean steady state, e.g. if protein A activates protein B and B represses A, then when A is active B is turned ON, which turns A OFF and then turns B OFF and re-establishes the ON state for A, etc. With this modified simulation procedure, in this example A would turn B ON at *τ*_1_, then the negative feedback between B and A would become active at *τ*_2_ and lock A permanently to the OFF state (see [41]). As we can see on Figure 2, this method captures the slow negative feedback between ERK and SOS-1, with very limited additional computation cost.

### CNORdt: Boolean logic for time course data

Steady state and multiple steady states methods are useful first approximations to capture the dynamic behavior of a system when limited time resolved data is available. However, when time courses are available, we can get further insight by using methods that can fit such data. *CNORdt* (for Cellular Network OptimizeR discrete time) fits time course data using a synchronous updating scheme for the simulation (see equation 4), together with an additional model parameter, which defines the time step of the Boolean synchronous simulation. In a synchronous updating scheme, all nodes are updated simultaneously at each iteration of the simulation algorithm, such that the state of each node at time t depends only on the state of its parents at time t-1 [17]. The scaling parameter stretches the time courses obtained by Boolean synchronous update simulation to match the data as closely as possible. This approach captures behaviors such as oscillations, transients and feedbacks, provided that they can be fitted with a single scaling parameter across all reactions. Looking at our toy example (Figure 2), we can see that *CNORdt* accurately reproduces the transient behavior of ERK activation and the oscillatory behavior of NF*κ*B. Since *CNORdt* still trains a Boolean logic model (i.e. only the structure of the model is optimized), with only one additional parameter, the training stays relatively simple and computationally efficient.

### CNORfuzzy: constrained fuzzy logic at steady-state

A main limitation of Boolean logic models is that they are limited to ON/OFF representations of the activation levels of species in a model. This means that subtle effects and partial activations such as the activation of p38 in Figure 2 cannot be captured. Such phenomena require a continuous representation of nodes states, which is possible using fuzzy logic models as introduced in [35] and implemented in the MATLAB package CellNOpt-cFL. In *CNORfuzzy*, the relationships between nodes are defined as transfer functions linking continuous values of the inputs of each gate to continuous values of the outputs of each gate:

(5) $$\begin{array}{c} x_{i}(t+1)=\hat{B_{i}}(x_{i1}(t),x_{i2}(t),\ldots ,x_{i1}(t))\;\;\;\varepsilon [0,1],\;\;i=1,2,\ldots ,N \end{array}$$

In eq. 5, the Boolean function from eq. 4 is replaced by a transfer function $\hat{B_{i}}$ that maps the continuous value (bounded between 0 and 1) of input nodes at time t to continuous values of an output node *x*_*i*_ at time *t* + 1. In our implementation, transfer functions are limited to a defined set of Hill functions, hence the use of the term “constrained” fuzzy logic. Using normalized Hill functions ensures the consistency between the fuzzy logic values and the Boolean logic values when species are set to the extreme values of 0/1, and limits the number of parameters to be trained for each gate [35]. Training and simulation of the model in *CNORfuzzy* is very similar to the Boolean steady state optimization in *CellNOptR*. The difference is that we need to train both the topology of the model and the parameters of the transfer function associated with each gate. Given the added complexity of the optimization step, it is followed by refinement and reduction steps that fine-tune the parameters of the transfer functions and reduce the complexity of the model topology (see [35]). As we can see on Figure 2, *CNORfuzzy* accurately captures the partial activation of p38, as well as the activation of ERK and, to some extent, the activation of NF*κ*B. However, being a steady-state method, it is unable to capture ERK inactivation and NF*κ*B oscillations.

### CNORode: logic-based ordinary differential equations

*CNORode* (for ordinary differential equations) further refines the handling of time and state through a continuous representation of both variables. This is achieved by deriving a set of ordinary differential equations (ODEs) for each model species:

(6) $$\begin{array}{c} {\dot{x}}_{i}=\frac{1}{\tau_{i}}({\bar{B}}_{i}(x_{i1},x_{i2},\ldots ,x_{i1})-x_{i1})\;\;\;\varepsilon [0,1],\;\;i=1,2,\ldots ,N \end{array}$$

In equation 6, the Boolean updating function is replaced by a continuous activation function ${\bar{B}}_{i}$ for the production of *x*_*i*_ and a first order decay term, divided by a time constant *τ*_*i*_. For each species in the Boolean logic network, the ODE derived satisfies the condition that if the input of the gate to that species are Boolean (i.e. when species states tend to the limit 0 or 1), then the ODE for the species considered returns a value that is consistent with the value returned by the corresponding Boolean logic gate. The formalism used to derive the logic based ODEs was developed by [36] and is also implemented in the MATLAB toolbox Odefy [31]. For the optimization, *CNORode* generates a file that takes both discrete inputs that define the structure (optimized using one of the other above-mentioned methods) and continuous input values that correspond to the parameters of the ODEs (that *CNORode* aims to optimize). *CNORode* then trains the parameters of the equations to fit the data, using a choice of two stochastic, global optimization algorithms (a genetic algorithm or Scatter Search [38], as stated above). We can see in Figure 2 that *CNORode* accurately captures all of the dynamic features of the system at hand, i.e. the negative feedback between ERK and SOS-1, the negative feedback loop between NF*κ*B and I*κ*B, and the partial activation of p38 upon EGF and TNF*α*combined stimulations.

However, compared to the methods previously mentioned, this method requires: (i) the optimization of more parameters, therefore limiting the scalability, and (ii) the availability of detailed time resolved data.

### Case study: application of *CellNOptR* to a study of signaling in liver cancer

We illustrate the Boolean 2 steady-states *CellNOptR* method by applying it to a real data set. We use phosphorylation measurements (subset of the data in [1]) obtained from a human hepatocellular carcinoma cell line (HepG2) at 30 and 3 hours after perturbation with combinations of selected small molecule inhibitors. The experiment was designed to study the early and late response of to multiple inducers of inflammation, innate immunity and proliferation (see Additional file 1 for a full description of the readouts and perturbations; note that species are referred to in capital letters if the Uniprot identifier is used and in small letters if a colloquial/collective name is used). We use one of the variants of the Prior Knowledge Networks (PKN) that was used to analyze the data from [1] at 30 minutes in [11]. We extend the previous analysis [11] to include the data from [1] at 3 hours (see supplements).

As described, *CellNOptR* first pre-processed the PKN according to the data. Once compressed and expanded, the model contains 109 interactions. After training at *τ*_1_, between 24 and 27 edges are selected (based on 3 separate optimization runs, see Additional files 2 and 3), leading to an average training score of 0.031 (vs 0.066 for an empty model and 0.084 for the starting PKN-derived model). In this case, the empty model performs better than the starting PKN-derived model because many data points are close to 0, implying that many edges from the starting network are probably not functional in the context under investigation. Therefore, not turning any node ’ON’ actually achieves a better score than including all the edges (of which the vast majority are activating). After training at *τ*_2_, between 3 and 7 additional edges are selected, leading to an average optimization score of 0.094 (compared to an average of 0.124 if random edges are selected). Additional file 4: Figure S1 contains an example of a trained model and the corresponding data fit. We observe that the training does improve the fit of the model to data significantly at both time points compared to the starting PKN (t test p value <6.10−6 for *τ*_1_ and 0.03 for *τ*_2_). The improvement at *τ*_2_ is not as drastic as the one at *τ*_1_, likely because the PKN was designed for early events and therefore might not include all necessary prior knowledge edges to capture events happening at later times.

**Figure 3:**
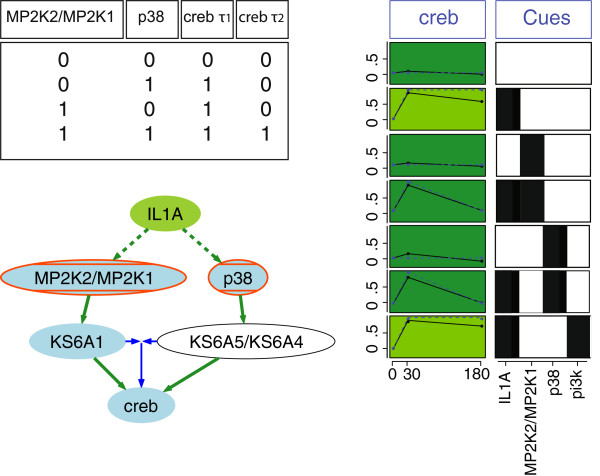
**Subset of the results of a *CellNOptR *analysis on two time-point data from human hepatocellular carcinoma cells.** The data consists of phospho-proteomic measurements of 16 proteins in response to multiple inducers of inflammation, innate immunity and proliferation, applied in combination with selected small molecule inhibitors [1]. This figure shows a simplified version of a small subset of the trained model (blue nodes=measured, green=stimulated, red=inhibited; green edges=picked at *τ*_1_, blue edges=picked at *τ*_2_), along with the data associated with the creb node (right, solid black line), overlaid with the simulation results (dashed blue line) for a selected set of conditions. The background color indicates the goodness of fit of simulation results to data. We can see that the model captures the behavior of creb accurately: creb increases at *τ*_1_ if either MP2K2/MP2K1 or p38 are activated (in this case, because both are downstream of IL1A, they are both activated in the absence of inhibitors and presence of IL1A). This activation is maintained if both MP2K2/MP2K1 and p38 are activated, and is lost at *τ*_2_ (180 minutes) if only one of them is activated (i.e. in this case if either is inhibited). This behavior is captured in the model by selecting an OR gate from MP2K2/MP2K1 and p38 to creb at *τ*_1_, and an AND gate at time *τ*_2_.

Nonetheless, the resulting trained models recapitulate some important behaviors. For example, it correctly captures a context-specific decrease in creb at *τ*_2_ (see Figure 3). The creb measurements increase at *τ*_1_ upon IL1A stimulation but this stimulated state is sustained at *τ*_2_ only if the signals going through KS6A1 (p90RSK) and KS6A4/KS6A5 (msk1/msk2, which are indirectly stimulated by IL1A) are both present (i.e. whereas an OR gate between these two branches accurately captures the increase of creb at *τ*_1_, an AND gate better captures the behavior at *τ*_2_). If there is an inhibition in either of these branches, creb does get activated at *τ*_1_ but then decreases at *τ*_2_. This means that for the creb signal to be maintained at *τ*_2_, the presence of both KS6A1 and KS6A4/KS6A5 is required. Such a behavior could, for example, be explained by a constitutive dephosphorylation of creb that can only be counteracted by the presence of both signals from KS6A1 and KS6A4/KS6A5. Sustained versus transient phosphorylation of creb following stimulation of the same receptor (NMDA) was observed in neurons and was shown to depend on the activity of the phosphatase Calcineurin [42]. This type of behavior is particularly relevant when studying diseases such as cancer. Indeed, sustained activation of transcription factors such as creb which are normally tightly regulated through transient phosphorylation has been proposed to play a role in oncogenesis [43]. This illustrates how our method captures dynamics occurring at different time scales, using relatively large scale models and only two time points. These features could not be captured by a one time point steady-state approach.

### Providing a user friendly interface with *CytoCopteR*

**Figure 4:**
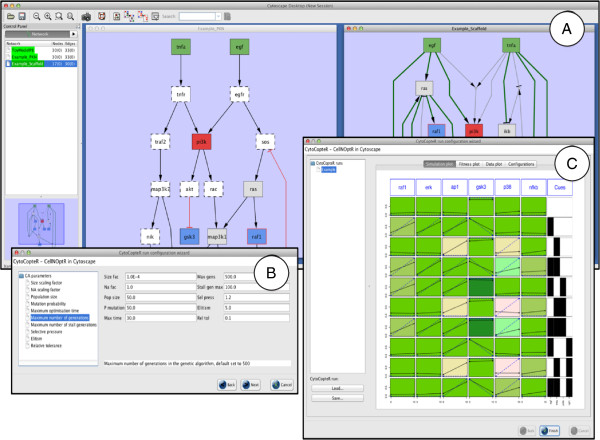
**Screenshot of *CytoCopteR*, the Cytoscape plugin for ***CellNOptR***.** Users can load or build a network in Cytoscape and load a matching data set in the MIDAS format, i.e. a CSV file with a row for each condition/time combination, a “TR:” column for each stimuli/inhibitor (0=absent,1=present) and for each readout a “DA:” column (time) and a “DV:” column (measurement). *CytoCopteR* annotates the original network with an overlaid color code on the edges and nodes (see subfigure **A**, left) reflecting the experimental (stimulated, inhibited, measured) and pre-processing (compressed or not) status for the nodes. Users then train the model to data, currently using the Boolean steady-state implementation in *CellNOptR*. The parameters for the training can be changed through explicit panels such as the one on subfigure **B**. Results of the pipeline are reported as in *CellNOptR*, via a graph displaying experimental and simulated data overlaid (see panel **C**), plots of the evolution of fit during the training process and diverse information of the training process (not shown). Furthermore, the scaffold network (after compression and expansion of the original network) is represented as a cytoscape network, with the same overlaid color code (see panel subfigure **A**, right) and weighting the edges according to their presence in the family of models retrieved.

Researchers who generate the kind of biochemical data that is amenable to logic modeling might not be familiar with R. Hence, we provide an intuitive and easy to learn graphical user interface (GUI) to our methods through a Cytoscape plugin, *CytoCopteR*. This results in a point and click interface to our methods where users can run the same steps as they would using an R script but without having to write any code (see Figure 4). Given that this is a front-end to the R algorithms, consistency is ensured between the results obtained through the GUI and those obtained through corresponding scripts. This arrangement also enables continued development of the methods and implementation in a single platform (R).

### Strengths of the *CellNOptR* modelling platform

**Figure 5:**
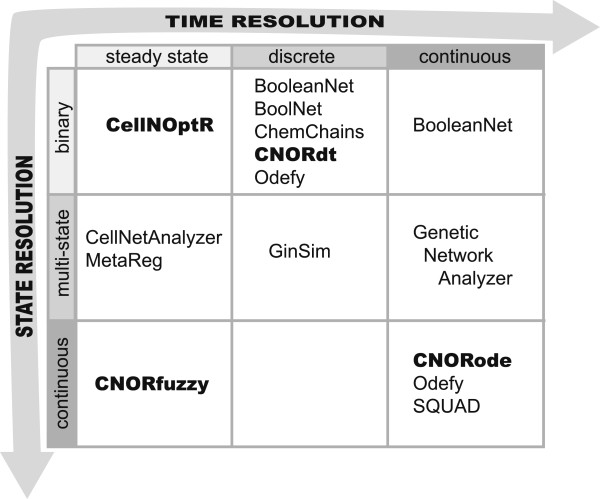
**Comparison with other softwares for logic modeling. ***Adapted from*[19]. These methods can be distinguished by their treatment of state and time. CellNetAnalyzer uses steady state analysis of logic models to better understand signaling and regulatory networks [24]. MetaReg [25] defines the prior knowledge of a system as a multi-state probabilistic model that can be simulated and visualized. BooleanNet [26] allows for synchronous and asynchronous simulations (as well as mixed approaches) and can also facilitate piecewise linear differential equations for a more detailed time resolution. BoolNet [27] also allows for synchronous and asynchronous simulations, and includes functionalities to deal with probabilistic Boolean networks where multiple transition functions can be chosen for each node. ChemChains is a software suite that allows for synchronous and asynchronous updating [28]. GINsim offers a suite of simulations methods that incorporates a graph editor as well as various tools to explore state transitions [29]. SQUAD [30] and Odefy [31] create continuous systems from logic models. Genetic Network Analyzer is a platform for modeling genetic regulatory networks, using piecewise linear models to model continuous processes [32]. *CellNOptR* and its extensions (*CNORfuzzy*, *CNORdt* and *CNORode*) cover steady state discrete and continuous modeling in both state and time. Note that because multi-state is a generalization of binary, in principle all methods that handle multi-states also handle binary.

A range of tools exists for manipulating, creating and simulating logic models (see Figure 5 for a more in depth description). *CellNOptR* differs in that it focuses on providing a method to systematically train models to data. This is an essential feature because, by leveraging imperfect and incomplete prior knowledge and dedicated signaling data, it builds and simulates models that are fitted to the data (thus cell type and context specific) and achieves a higher predictive power [11]. This has proven useful, for example, to obtain cell specific models that reveal different wiring between cell-types, by training a network separately to data from different cell types [44]. Because this is achieved with a simple modeling framework, one can investigate large networks with relatively sparse data compared to other formalisms for modeling of signaling networks [12,17,45,46]. The mechanistic insight that can be gained is higher than in purely data driven models, which can only capture relationships between perturbed and observed variables (models built using our pipeline also include intermediates).

The method described in [11], i.e. the Boolean single steady-state implementation, was previously implemented in a MATLAB toolbox, *CellNOpt*. *CellNOpt* was also extended to constrained fuzzy logic as described in [35]. However, the Boolean 2 steady-states, discrete time and logic ODE variants are unique to the R implementation presented here. This extension is an essential strength of *CellNOptR* since the toolkit presented here uniquely covers a wide variety of different logic modeling methods adapted to different experimental scenarios and modeling goals, all available within the same training framework.

### Future developments

We consider the existing version of *CellNOptR* as a robust and flexible starting point for multiple developments. For example, we are exploring alternative methods for data normalization. The core of CellNOpt is the training to data, and we are exploring multiple strategies for this, including deterministic methods such as integer linear programming [47,48] and answer set programming [49], metaheuristics [38], and probabilistic frameworks [25].

While CellNOpt already covers multiple logic formalisms, we are exploring other variants, in particular asynchronous simulation schemes for the CNORdt extension. This could lead to different results to those obtained with the synchronous scheme, which could be particularly insightful when handling single cell time course data. Given the stochastic nature of an asynchronous update scheme, when using population averaged data (as has been the case so far) one needs to run the simulation many times to generate a set of trajectories from which a consensus can be obtained. This is considerably more demanding computationally, and is not likely to provide additional insight in most simple cases. In the case of the example toy model from Figure 2, asynchronous simulation where activation rules are fired at random did not provide additional information (see Additional file 5). Different conclusions might be obtained when using larger networks with more complicated feedback, or when information is available regarding the order of firing of different activation rules. We are therefore currently working on making alternative simulation schemes available, as well as faster versions of those (mostly based on C implementations).

Another main area of development is the integration of data-driven reverse engineering tools to find links missing in the starting network [15]. A main strength of *CellNOptR* is also one of its weaknesses: the optimization is constrained by the PKN. To address this limitation, the plugin CNOFeeder allows to propose candidate links based on areas of the data that are poorly captured by the trained model, using multiple reverse engineering methods [50].

Finally, we are working to make communication and exchange of data and models to and from *CellNOptR* both easy and consistent. A requirement towards this goal is compliance to standards. We are currently working on using the Systems Biology Graphical Notation (SBGN, http://www.sbgn.org/) for visualization of models using a standard set of symbols. Towards this end, we have developed the Cytoscape plugin CySBGN (http://sourceforge.net/projects/cysbgn/), that we plan to integrate with CytoCopter. Furthermore, we are developing, as part of the CoLoMoTo initiative (http://www.colomoto.org) a qualitative extension for SBML (http://sbml.org/Community/Wiki/SBML_Level_3_Proposals/Qualitative_Models), SBML-Qual. This extension will allow not only a smooth exchange of our models with other logic modeling tools with complementary features, but also automatic access to resources for prior knowledge information that are compliant with it, such as path2models (http://www.ebi.ac.uk/biomodels-main/path2models). The *CellNOptR* project is in continuous development and users can find updates on the project website (http://www.cellnopt.org).

## Conclusions

Understanding signal processing in cells is an essential goal of biological research, not only for fundamental reasons but also for its implications and potential applications in disease contexts. Modeling approaches are particularly suited to this task because (i) signaling networks are complex systems assembled from the dynamic and context-dependent interactions of many components, and (ii) obtaining predictive as well as mechanistic insights is extremely valuable in this context. *CellNOptR* makes use of the complementarity between rich context specific biochemical data and imperfect/incomplete accumulated knowledge to build and train logic models.

*CellNOptR* models are constrained by previous knowledge but trained to data, making them both context and cell line specific, thereby providing enhanced predictive and mechanistic insights. A key strength of the toolkit formed by *CellNOptR*, *CNORdt*, *CNORode* and *CNORfuzzy* is that it covers multiple logic modeling formalisms (Boolean steady-state, Boolean multiple steady-state, Boolean time courses through synchronous update, steady-state constrained fuzzy logic and continuous logic-based ODEs). This allows users to choose between those formalisms to best match the richness of their data and their modeling goals. We believe that this choice is greatly simplified by the availability of these methods in a common framework. One can also combine formalisms: for example, train a large network to data using the efficient multiple pseudo steady state method, and then convert the resulting sparser model into an ODE model and train it to time course data using *CNORode*.

Our toolkit is implemented in the free and open source R language and Cytoscape platform which benefit from a large user community and already come with a wide range of packages for biological data processing and analysis. Users should therefore be able to use *CellNOptR* as part of their own data processing pipeline, taking advantage of existing R/Bioconductor packages (e.g. for data normalization, visualization etc.) and developing their own custom-made functions as required. Finally, in order to make our methods more accessible to non-programmers, we provide a Cytoscape interface to the R implementation as a plugin, *CytoCopteR*.

## Availability and requirements

The main *CellNOptR* package is available on Bioconductor (http://www.bioconductor.org/packages/release/bioc/html/CellNOptR.html) as well as the CNORdt, CNORode and CNORfuzzy add-on packages. The *CytoCopteR* Cytoscape plugin is available on http://www.cellnopt.org and from the Cytoscape plugin manager. The simplest *CellNOptR* method (Boolean steady state) and the fuzzy logic methods are available in a MATLAB version of the toolbox, also available at http://www.cellnopt.org.

More details: 

- **Software name:***CellNOptR* (CellNetOptimizeR), with plug in packages *CNORdt* (CellNetOptimizeR discrete time), *CNORode* (ordinary differential equations) and *CNORfuzzy* (fuzzy logic), and Cytoscape plug in interface *CytoCopteR*.

- **Project home page:**http://www.cellnopt.org

- **Operating system(s):** platform independent

- **Programming languages:** R

- **Other requirements:** R (tested on 2.13 and above), Cytoscape 2.x

- **License:** GNU-GPL, version 3 except CNORfuzzy which is GNU-GPL version 2.

## Competing interests

The authors declare that they have no competing interests.

## Authors’ contributions

CT and TC wrote the *CellNOptR* package, AM wrote the *CNORdt* package, MKM and TC wrote the *CNORfuzzy* package, DH wrote the *CNORode* package, EG and MvI wrote the *CytoCopteR* plugin, JSR designed the project, and together with DAL provided guidance for the implementation. CT and JSR wrote most of the manuscript, with contributions from the other authors. All authors read and approved the final manuscript.

## Supplementary Material

Additional file 1 **Experimental setting for the HepG2 analysis.** HepG2 cells were stimulated with the above stimuli in combination with the above-mentioned inhibitors in different combinations. The 16 species mentioned here were then measured using a luminex assay at 30 minutes and 3 hours post stimulation, leading to a total of 136 samples. All species are mentioned with their Uniprot identifiers (capital letters) or common name where applicable (small caps letters). Click here for file

Additional file 2 **Summary of results from 3 independent trainings for the HepG2 example.** Frequency of selection of each edge in the scaffold model, across all models with a score within 10% of the best scoring model, summarized across 3 independent training runs. The top panel shows the summary for the edges at time 1and the bottom panel shows the equivalent for time 2. For time 1, 13 edges are consistently selected across most (> 80%) of the best performing model, and 24 edges are picked in over 60% of the trained models. A partial redundancy in the effect of some edges explains that a different combination of edges can be picked across different models with limited impact on their scores. At time 2 (lower panel), 5 edges are consistently selected across over 50% of the best scoring models. These lower numbers reflect the fact that the training at time 2 relies on a single trained model as a starting point for both the simulation and the edge search space. Therefore, the family of trained models obtained for each of the training runs explore different search spaces and have different initial conditions. Click here for file

Additional file 3 **Technical aspects of the HepG2 analysis.** This file provides additional information regarding this analysis, such as the parameters used etc. Click here for file

Additional file 4 **Example of results for the HepG2 real data application.** A. Previous knowledge network used for this analysis. B. Example of a trained model obtained in one of the optimization round, with a subset of the simulation results obtained with this network (C). For the networks the color codes are as follows: nodes: green=stimulated, red=inhibited, blue=measured, blue with red stroke=measured and inhibited, dashed stroke=compressed; edges (in the trained model in panel B): green=selected at time 1, blue=selected at time 2, grey=not selected in the trained model. In panel C, black continuous lines=data, dashed blue lines=simulation results obtained with the model in B. The background color reflects the goodness of fit of the model to data: green= the chosen Boolean value is closer to the data than the opposite Boolean value (the darker, the closer), red= the chosen Boolean value is further from the data than the opposite Boolean value (the darker, the further). Click here for file

Additional file 5 **Exploration of an asynchronous updating scheme for the CNORdt extension.** This figure shows the results obtained by training the toy model to data as in Figure 2 but using an asynchronous updating scheme with random firing order of the activation rules, in development for the CNORdt extension. We can see that asynchronous updating adds no new information that is applicable to training the model to data, in this case. For the same conditions as Figure 2, the asynchronous plots show the fraction of simulations (out of 100) where each specified node is switched on (y-axis) after each update of the network (x-axis). The error bars show ± 1 standard deviation of the 100 simulations at each iteration (only 1 in every 10 displayed). In the case of the above model, negative feedback causes oscillations and oscillating nodes average ∼ 0.5. All other nodes stabilize at 0/1. The synchronous plots use the same simulator described in the main text under CNORdt, where all nodes are updated at the same time t according to the state of their input nodes at t-1. Click here for file
