# Supplementary material for: CellNOptR: a flexible toolkit to train protein signaling networks to data using multiple logic formalisms
Source: BMC Syst Biol. 2012 Oct 18;6:133. doi: 10.1186/1752-0509-6-133 (PMC3605281; doi:10.1186/1752-0509-6-133)
Supplement: Additional file 1 — Experimental setting for the HepG2 analysis. HepG2 cells were stimulated with the above stimuli in combination with the above-mentioned inhibitors in different combinations. The 16 species mentioned here were then measured using a luminex assay at 30 minutes and 3 hours post stimulation, leading to a total of 136 samples. All species are mentioned with their Uniprot identifiers (capital letters) or common name where applicable (small caps letters). [file 1752-0509-6-133-S1.pdf]

| | |
|---|---|
| Measurements | AKT1/AKT2/AKT3 (akt)<br>MK01/MK03 (erk1/erk2)<br>GSK3A/GSK3B<br>IKBA<br>JNK1/2 (MK08/MK09)<br>MK11/MK12/MK13/MK14 (p38)<br>KS6B1 (p70s6)<br>KS6B1 (p90rsk)<br>STAT3<br>JUN<br>CREB1/ATF4/CREB3/CREB5 (creb)<br>H31T/H33 (histH3)<br>HSPB1 (hsp27)<br>IRS1<br>MP2K2/MP2K1 (mek1/mek2)<br>P53 |
| Stimuli | IFNG<br>TNFA<br>IL1A<br>IL6<br>IGF1<br>TGFA<br>lps |
| Inhibitors | MP2K2/MP2K1 (mek1/mek2)<br>MK11/MK12/MK13/MK14 (p38)<br>PK3CA/PK3CB/PK3CG/P85A/P85B/P55G/PI3R4/PI3R5/PI3R6 (pi3k)<br>MTOR<br>GSK3A/GSK3B<br>MK08/MK09<br>IKKA/IKKB/NEMO (ikk) |
