# Supplementary figures and images for: CellNOptR: a flexible toolkit to train protein signaling networks to data using multiple logic formalisms
Source: BMC Syst Biol. 2012 Oct 18;6:133. doi: 10.1186/1752-0509-6-133 (PMC3605281; doi:10.1186/1752-0509-6-133)

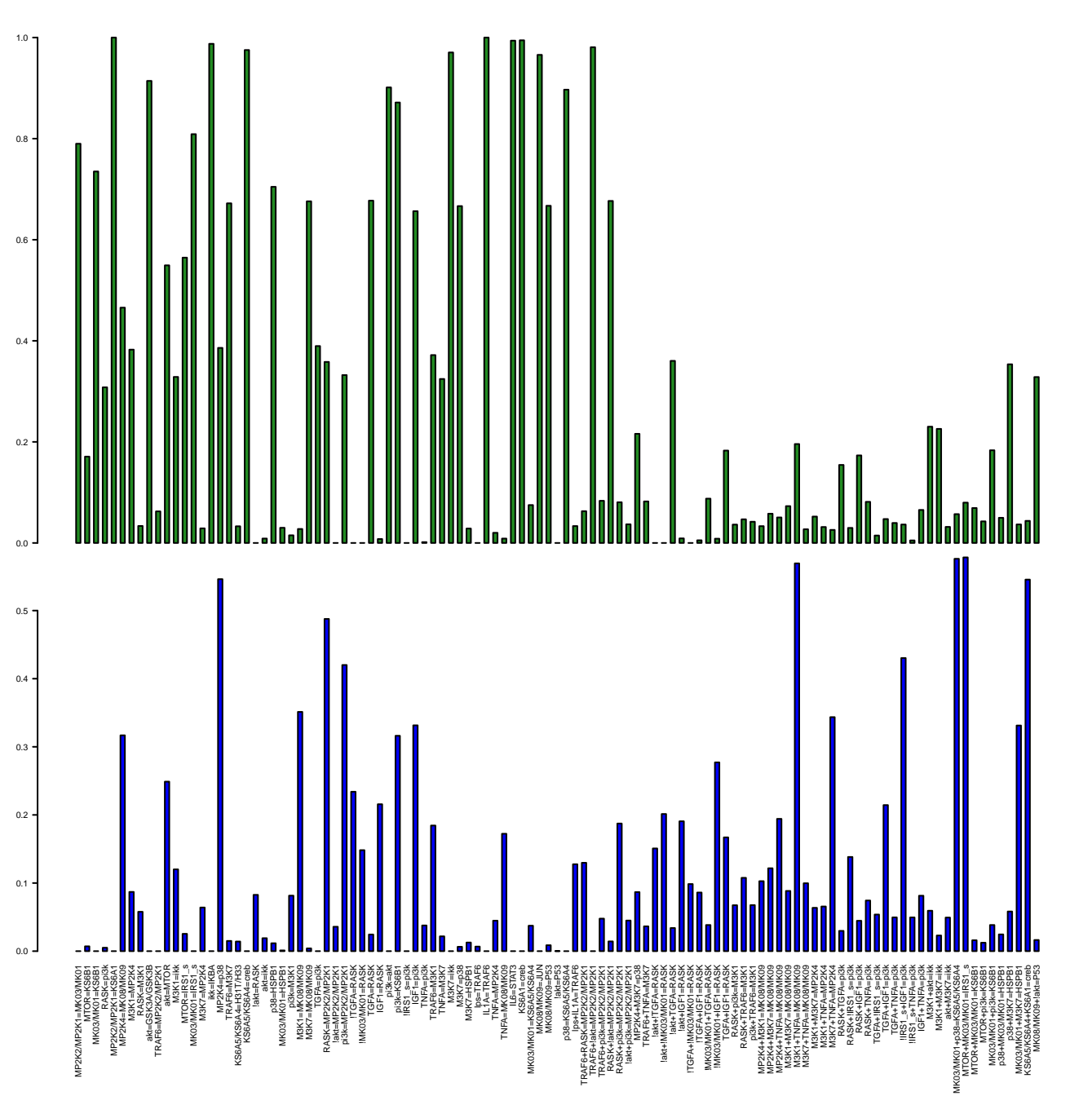

Supplement: Additional file 2 — Summary of results from 3 independent trainings for the HepG2 example. Frequency of selection of each edge in the scaffold model, across all models with a score within 10% of the best scoring model, summarized across 3 independent training runs. The top panel shows the summary for the edges at time 1and the bottom panel shows the equivalent for time 2. For time 1, 13 edges are consistently selected across most (> 80%) of the best performing model, and 24 edges are picked in over 60% of the trained models. A partial redundancy in the effect of some edges explains that a different combination of edges can be picked across different models with limited impact on their scores. At time 2 (lower panel), 5 edges are consistently selected across over 50% of the best scoring models. These lower numbers reflect the fact that the training at time 2 relies on a single trained model as a starting point for both the simulation and the edge search space. Therefore, the family of trained models obtained for each of the training runs explore different search spaces and have different initial conditions. [file 1752-0509-6-133-S2.pdf]
