# Supplementary material for: CellNOptR: a flexible toolkit to train protein signaling networks to data using multiple logic formalisms
Source: BMC Syst Biol. 2012 Oct 18;6:133. doi: 10.1186/1752-0509-6-133 (PMC3605281; doi:10.1186/1752-0509-6-133)
Supplement: Additional file 3 — Technical aspects of the HepG2 analysis. This file provides additional information regarding this analysis, such as the parameters used etc. [file 1752-0509-6-133-S3.pdf]

Technical aspects of the HepG2 analysis

The model training was repeated three times independently. Each training was run until the best score was stable over 100 generations of the genetic algorithm. For the normalization we used all default parameters except for the threshold for detection level which was set to 0. This is because some signals have very low values at time 0 but get very strongly stimulated at time 1 and decrease to their initial value at time 2. Given that significant levels are observed at time 1 we reasoned that the low values at time 0 and time 2 were not the result of an experimental problem but reflected the fact that the level of these phosphoproteins were indeed very low. The detection level parameter is introduced to exclude unreliable measurements. For example when a phosphoprotein measurement is always below the detection level of the instrument, then these measurements are set to 'NA' because otherwise the normalization procedure would artificially increase the small variations due to noise. In this case, we can be fairly confident that this would not happen since very low measurements will be correctly qualified as low in comparison to high measurements available for the same species. Failure to notice this would mean that all species going from very low to high will not be captured since the low starting or ending point would be missing. All other parameters were set to their default values.

Note that the training scores are different from those obtained in *Saez-Rodriguez et al. (MSB, 2009)* and *Morris et al., (Plos Comp. Bio., 2011)* because these publications used, besides different networks, different parameters for the normalization of the data and only made use of the 30 minutes time point.
