# Supplementary material for: CellNOptR: a flexible toolkit to train protein signaling networks to data using multiple logic formalisms
Source: BMC Syst Biol. 2012 Oct 18;6:133. doi: 10.1186/1752-0509-6-133 (PMC3605281; doi:10.1186/1752-0509-6-133)
Supplement: Additional file 5 — Exploration of an asynchronous updating scheme for the CNORdt extension. This figure shows the results obtained by training the toy model to data as in Figure 2 but using an asynchronous updating scheme with random firing order of the activation rules, in development for the CNORdt extension. We can see that asynchronous updating adds no new information that is applicable to training the model to data, in this case. For the same conditions as Figure 2, the asynchronous plots show the fraction of simulations (out of 100) where each specified node is switched on (y-axis) after each update of the network (x-axis). The error bars show ± 1 standard deviation of the 100 simulations at each iteration (only 1 in every 10 displayed). In the case of the above model, negative feedback causes oscillations and oscillating nodes average ∼ 0.5. All other nodes stabilize at 0/1. The synchronous plots use the same simulator described in the main text under CNORdt, where all nodes are updated at the same time t according to the state of their input nodes at t-1. [file 1752-0509-6-133-S5.pdf]

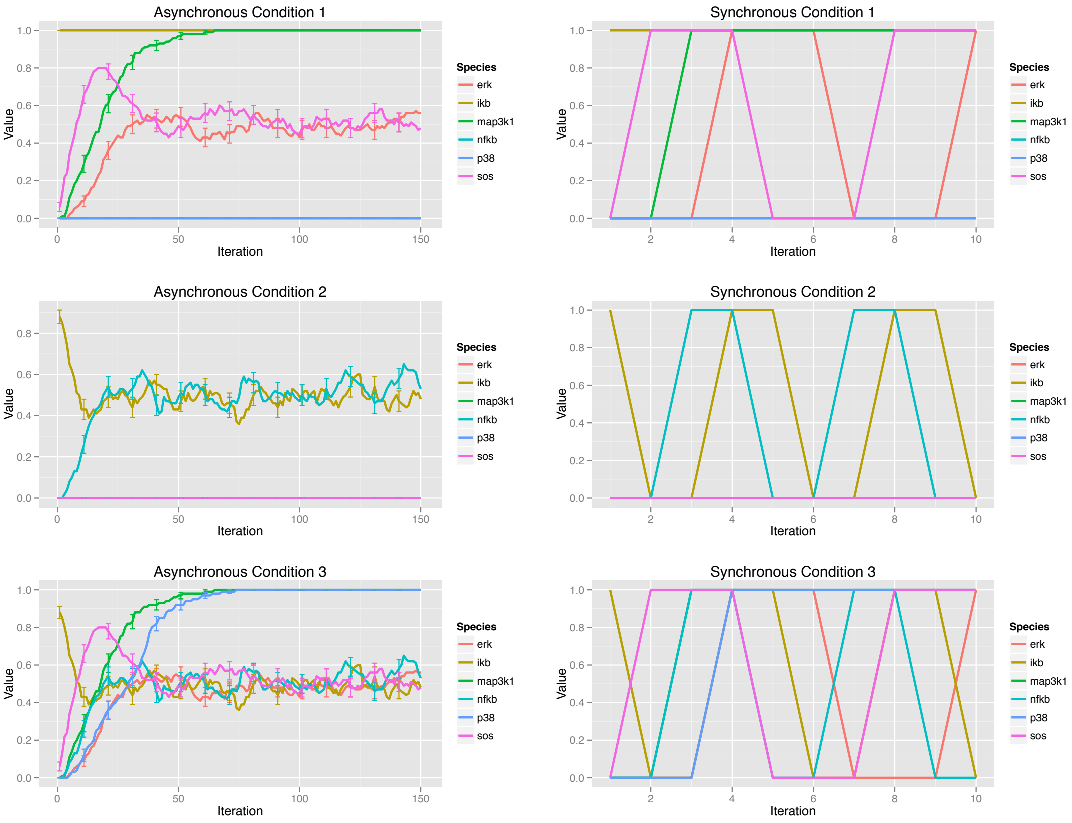
Asynchronous Condition 1
Synchronous Condition 1
Asynchronous Condition 2
Synchronous Condition 2
Asynchronous Condition 3
Synchronous Condition 3
Value
Iteration
Species
erk
ikb
map3k1
nfkb
p38
sos
